# Supplementary material for: Cryo-EM of cardiac AL-224L amyloid reveals shared features in λ6 light chain fibril folds
Source: bioRxiv. 2025 Jul 1:2025.06.25.661559. Preprint. [Version 2] doi: 10.1101/2025.06.25.661559 (PMC12236830; doi:10.1101/2025.06.25.661559)
Supplement: Supplement 1 [file NIHPP2025.06.25.661559v2-supplement-1.pdf]

EXTENDED DATA

Characteristics	AL-224	Reference range
Demographic features		
Gender	F	
Age, years	63	
Age at death, years	63	
Cause of death	sudden death	
Echocardiographic features		
Interventricular septal thickness (mm)	11	
Ejection fraction, %	60	
Laboratory parameters		
Bone marrow plasma cells, %	n/a	
LC restriction	λ	
SIFE	IgG+ λ FLC	
FLC κ	9.20	3.3–19.4
FLC λ	175.00	5.7–26.3
FLC ratio	0.05	0.26–1.65
Abnormal HLC, isotype	IgG	
IgG HLC κ	1.6	3.84–12.07
IgG HLC λ	8.49	1.91–6.74
IgG HLC ratio	0.19	1.12–3.21
UIFE	λ FLC	
BNP, pg/ml	126	0-176
Troponin I, ng/ml	0.046	<0.033
Serum creatinine, mg/dl	0.91	0.7–1.3
eGFR, ml/min/1.73 m^2	71	
24-h urine protein, mg	469	
Alkaline phosphatase, U/l	127	25–100
Organ involvement		
Cardiac	+	
Soft tissue	+	
Congo red positive histology		
Fat pad aspirate	+	
Bone marrow biopsy	+	

Table S1. **Demographic, clinical and laboratory characteristics of case AL-224 with AL amyloidosis at initial evaluation.** Abbreviations: BNP, B-type natriuretic peptide; eGFR, estimated glomerular filtration rate; FLC, free light chain; HLC, heavy light chain; Ig, immunoglobulin; LC, light chain; SIFE, serum immunofixation electrophoresis; UIFE, urine immunofixation electrophoresis.

Accession	Description	Coverage [%]	# PSMs	# Unique Peptides	# AAs	MW [kDa]
P12883	Myosin-7 OS=Homo sapiens OX=9606 GN=MYH7 PE=1 SV=5	76	841	133	1935	223
P13533	Myosin-6 OS=Homo sapiens OX=9606 GN=MYH6 PE=1 SV=5	50	521	3	1939	223.6
AL-224L	Amyloidogenic LC sequence AL-224L OS=Homo sapiens	74	373	40	218	23.3
Q8WZ42	Titin OS=Homo sapiens OX=9606 GN=TTN PE=1 SV=4	7	257	224	34350	3813.7
P62736	Actin, aortic smooth muscle OS=Homo sapiens OX=9606 GN=ACTA2 PE=1 SV=1	70	187	39	377	42
P12882	Myosin-1 OS=Homo sapiens OX=9606 GN=MYH1 PE=1 SV=3	25	157	4	1939	223
P35609	Alpha-actinin-2 OS=Homo sapiens OX=9606 GN=ACTN2 PE=1 SV=1	44	112	42	894	103.8
P60709	Actin, cytoplasmic 1 OS=Homo sapiens OX=9606 GN=ACTB PE=1 SV=1	47	110	5	375	41.7
P06576	ATP synthase subunit beta, mitochondrial OS=Homo sapiens OX=9606 GN=ATP5F1B PE=1 SV=3	70	99	58	529	56.5
Q14896	Myosin-binding protein C, cardiac-type OS=Homo sapiens OX=9606 GN=MYBPC3 PE=1 SV=4	38	93	64	1274	140.7
P12111	Collagen alpha-3(VI) chain OS=Homo sapiens OX=9606 GN=COL6A3 PE=1 SV=5	16	76	53	3177	343.5
P25705	ATP synthase subunit alpha, mitochondrial OS=Homo sapiens OX=9606 GN=ATP5F1A PE=1 SV=1	54	63	41	553	59.7
P17540	Creatine kinase S-type, mitochondrial OS=Homo sapiens OX=9606 GN=CKMT2 PE=1 SV=2	39	57	30	419	47.5
P04004	Vitronectin OS=Homo sapiens OX=9606 GN=VTN PE=1 SV=1	25	52	17	478	54.3
Q08043	Alpha-actinin-3 OS=Homo sapiens OX=9606 GN=ACTN3 PE=1 SV=2	14	52	3	901	103.2
P06727	Apolipoprotein A-IV OS=Homo sapiens OX=9606 GN=APOA4 PE=1 SV=4	55	45	37	396	45.3
P02511	Alpha-crystallin B chain OS=Homo sapiens OX=9606 GN=CRYAB PE=1 SV=2	65	36	23	175	20.1
Q9Y2K3	Myosin-15 OS=Homo sapiens OX=9606 GN=MYH15 PE=1 SV=6	3	32	2	1926	222
P12109	Collagen alpha-1(VI) chain OS=Homo sapiens OX=9606 GN=COL6A1 PE=1 SV=3	19	29	20	1028	108.5

P48735	Isocitrate dehydrogenase [NADP], mitochondrial OS=Homo sapiens OX=9606 GN=IDH2 PE=1 SV=2	33	28	15	452	50.9
P07437	Tubulin beta chain OS=Homo sapiens OX=9606 GN=TUBB PE=1 SV=2	34	28	3	444	49.6
P68371	Tubulin beta-4B chain OS=Homo sapiens OX=9606 GN=TUBB4B PE=1 SV=1	38	28	4	445	49.8
P17661	Desmin OS=Homo sapiens OX=9606 GN=DES PE=1 SV=3	33	27	15	470	53.5
P12814	Alpha-actinin-1 OS=Homo sapiens OX=9606 GN=ACTN1 PE=1 SV=2	14	27	6	892	103
P55809	Succinyl-CoA:3-ketoacid coenzyme A transferase 1, mitochondrial OS=Homo sapiens OX=9606 GN=OXCT1 PE=1 SV=1	28	27	19	520	56.1
P54296	Myomesin-2 OS=Homo sapiens OX=9606 GN=MYOM2 PE=1 SV=3	15	23	19	1465	164.8
P21796	Non-selective voltage-gated ion channel VDAC1 OS=Homo sapiens OX=9606 GN=VDAC1 PE=1 SV=2	57	22	16	283	30.8
P12110	Collagen alpha-2(VI) chain OS=Homo sapiens OX=9606 GN=COL6A2 PE=1 SV=4	16	21	18	1019	108.5
P45880	Voltage-dependent anion-selective channel protein 2 OS=Homo sapiens OX=9606 GN=VDAC2 PE=1 SV=2	34	21	10	294	31.5
P02649	Apolipoprotein E OS=Homo sapiens OX=9606 GN=APOE PE=1 SV=1	34	21	15	317	36.1

Table S2. Top 30 master proteins identified with at least two unique peptides of heart-tissue extracted fibrils by LC-MS/MS.

Abbreviations: PSM, peptide-spectrum match; #AAs, number of amino acids in full-length protein; MW, molecular weight. The proteins are ranked according to the number of PSMs. Fibril-forming protein AL-224L (pink), collagen VI chains a1, a 2 and a 3 (green), and the amyloid signature proteins apoA-IV and apoE (orange) are highlighted. Serum amyloid P-component was detected in low abundance (#39, not shown). The data normalization using normalized spectral abundance factor (NSAF) values demonstrated AL-224L as the top identified protein (data not shown). To calculate the NSAF value for each protein, the spectral counts were divided by the protein length (PSM/#AAs) and the sum of all PSM/#AA values was divided by the number of all identified proteins as previously reported (Neilson KA, Keighley T, Pascovici, D, Cooke B, Haynes PA. (2013) Label-Free Quantitative Shotgun Proteomics Using Normalized Spectral Abundance Factors. Methods Mol Biol. 2013:1002: 205-222).

LC sub-family	PDB ID	ΔG, kcal/mol
λ3	9FAA	-33.7
λ3	9FAB	-33.4
λ3	9FAC	-34.2
λ3	8R47	-26.4
λ3	9EME	-25.5
λ1	7NSL	-26.6
λ1	6IC3	-24.2
λ3	6Z1O	-21.5
λ3	6Z1I	-20.4
λ6 AL55 renal	8CPE	-23.3
λ6 AL55 cardiac	6HUD	-20.2
**λ6 AL-224L cardiac**	**9OKA**	**-18.0**
λ6 germline	6HUD	-18.6
λ6 germline	9OKA	-22.1

Table S3. Solvation energy calculated for the available amyloid structures of AL LCs. ΔG values represent free energy change upon interface formation between two fibril layers, which was estimated using Protein Interfaces, Surfaces and Assemblies (PDBePISA) server, https://www.ebi.ac.uk/msd-srv/prot_int/cgi-bin/piserver. Bottom two rows list hypothetical values for the λ6-LC germline sequence placed in the amyloid structure of cardiac AL55 LC (PDB: 6HUD) or cardiac AL-224L (PDB: 9OKA). Mutations to restore the germline sequence were introduced using the Swiss-PdbViewer software, https://spdbv.unil.ch/ and the structure was energy-minimized to relieve steric clashes.

Sample	SCFA µM	Recovery µM	Recovery %
SCFA	19.97		
NEFA	0		
SCFA + NEFA	21.89	21.89	109.6
**Fr3**	**38.71**		
Fr3 + NEFA	36.75	36.75	95.0
Fr3 + SCFA	56.92	18.21	91.1
Fr3 + SCFA + NEFA	88.26	51.49	74.9

Table S4. Short chain fatty acid content in the amyloid-containing tissue extracts (fraction 3) measured by ELISA and assay validation.
Abbreviations: SCFA - short chain fatty acid; NEFA – non-esterified fatty acid; Fr3 – tissue-extracted fraction 3 used for cryo-EM analysis of amyloid. The experimental details are reported in Methods.

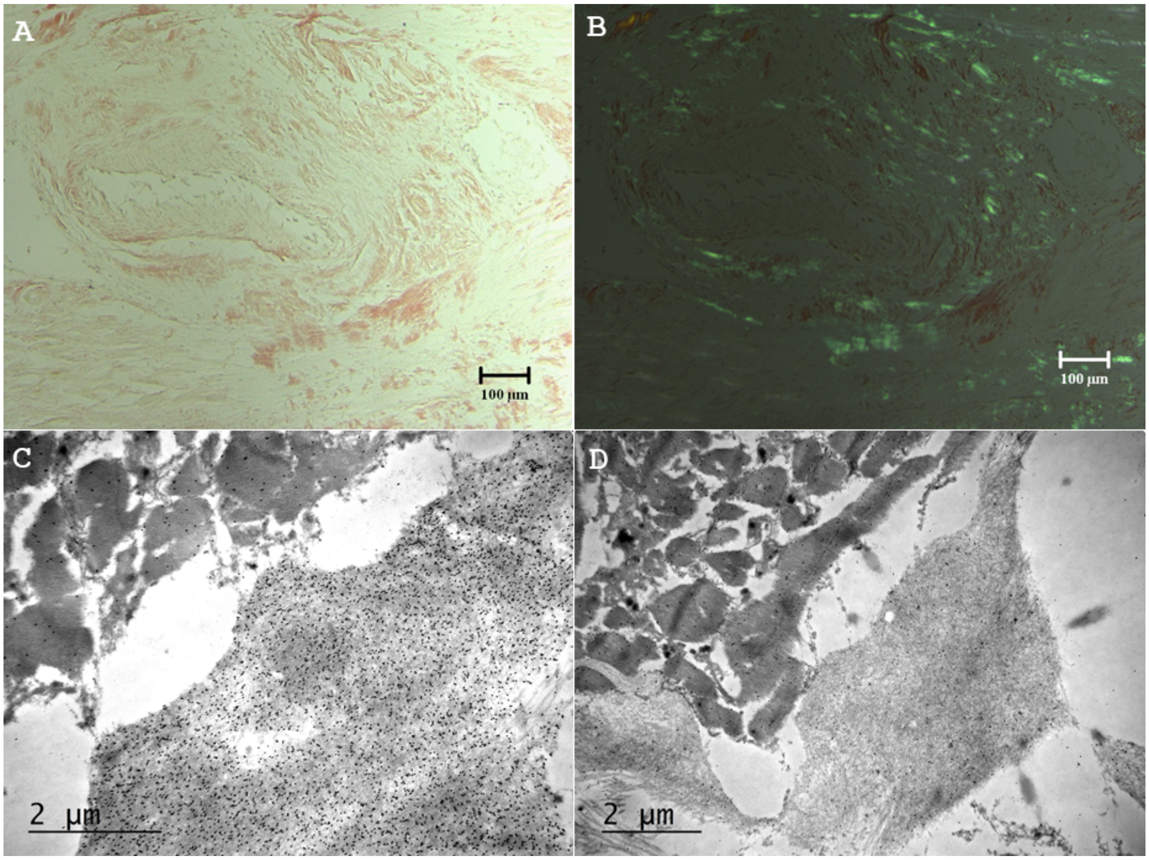

Figure S1. Histological analyses of the autopsied cardiac tissue in case AL-224.
A, Light microscopy images of Congo red-stained cardiac tissue indicate amyloid deposits. Original magnification ×100. **B**, Areas in panel A viewed by polarized microscopy show amyloid deposits with characteristic green birefringence. Original magnification ×100. **C, D**, Electron micrographs of autopsied cardiac tissue show haystack-like organization of amyloid fibrils in the extracellular space. Immunogold labeling demonstrates numerous electron dense deposits with antibody directed against immunoglobulin λ-LC (original magnification ×20,000, **(C)** and no immunoreactivity with antibody directed against immunoglobulin κ-LC (original magnification ×15,000, **(D)**.

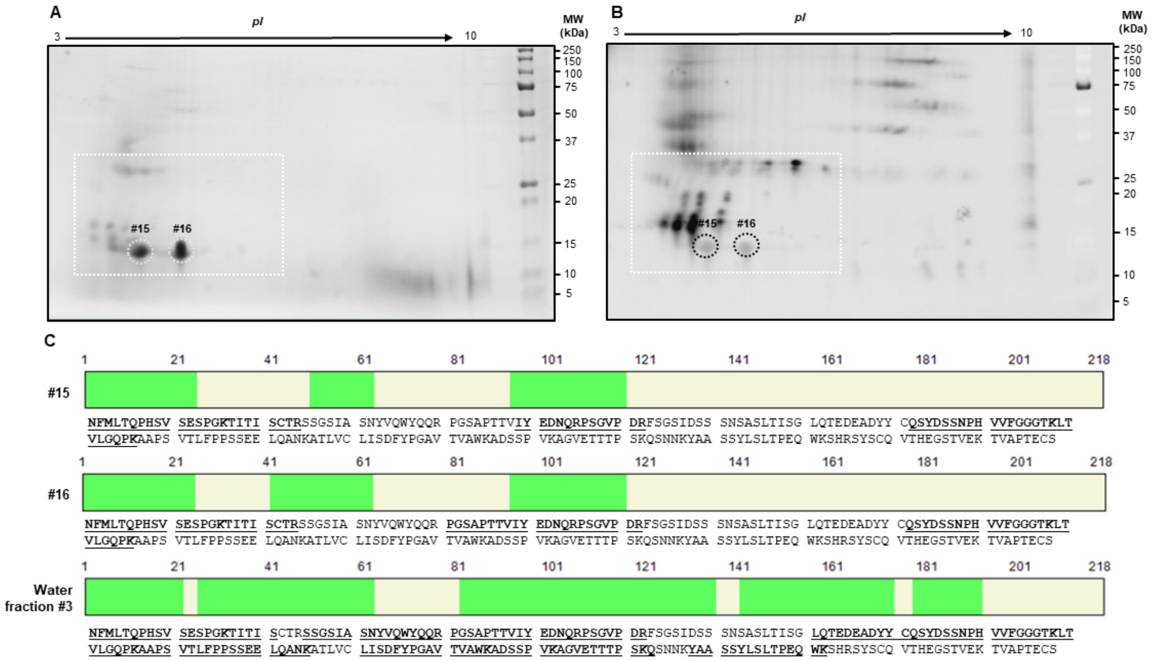

Figure S2. Analysis of AL-224 proteins in water fraction 3 of cardiac tissue fibril extract using 2D SDS-PAGE, 2D western blotting and LC-MS/MS.

A, Coomassie-stained gel and **B**, the corresponding 2D western blot probed with a primary anti-human λ light chains antibody. Two most prominent spots are circled and marked #15 and #16. These spots were excised and analyzed by LC-MS/MS, along with the entire water fraction 3. **C**, Amino acid sequence of AL-224L shows the position (in green) and sequence (underlined bold) of the peptides identified in spots #15, #16 and in the entire water fraction 3 (indicated).

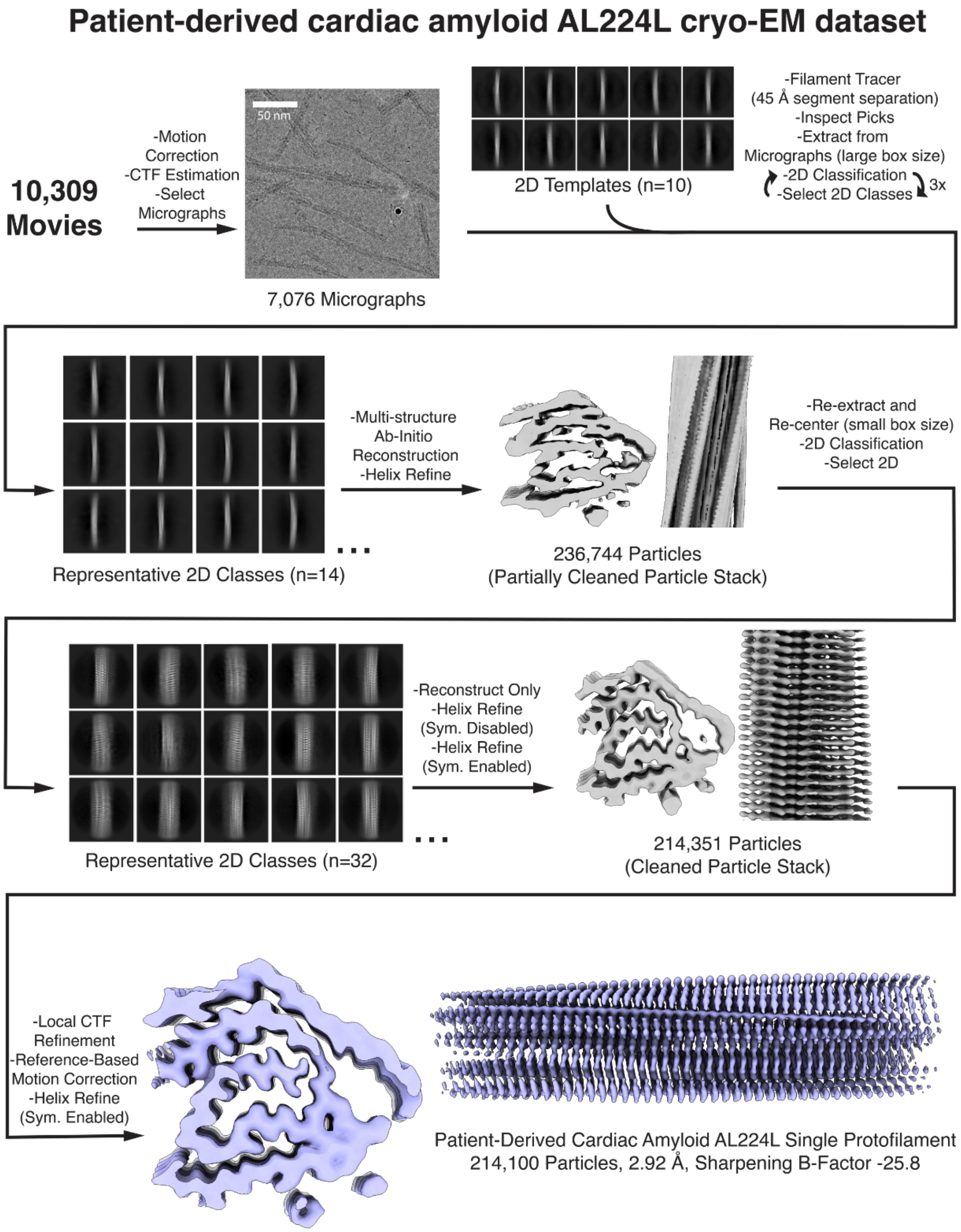

Figure S3. Cryo-EM data processing workflow.
General processing pipeline to generate an EM map of an AL-224L single-filament amyloid polymorph.

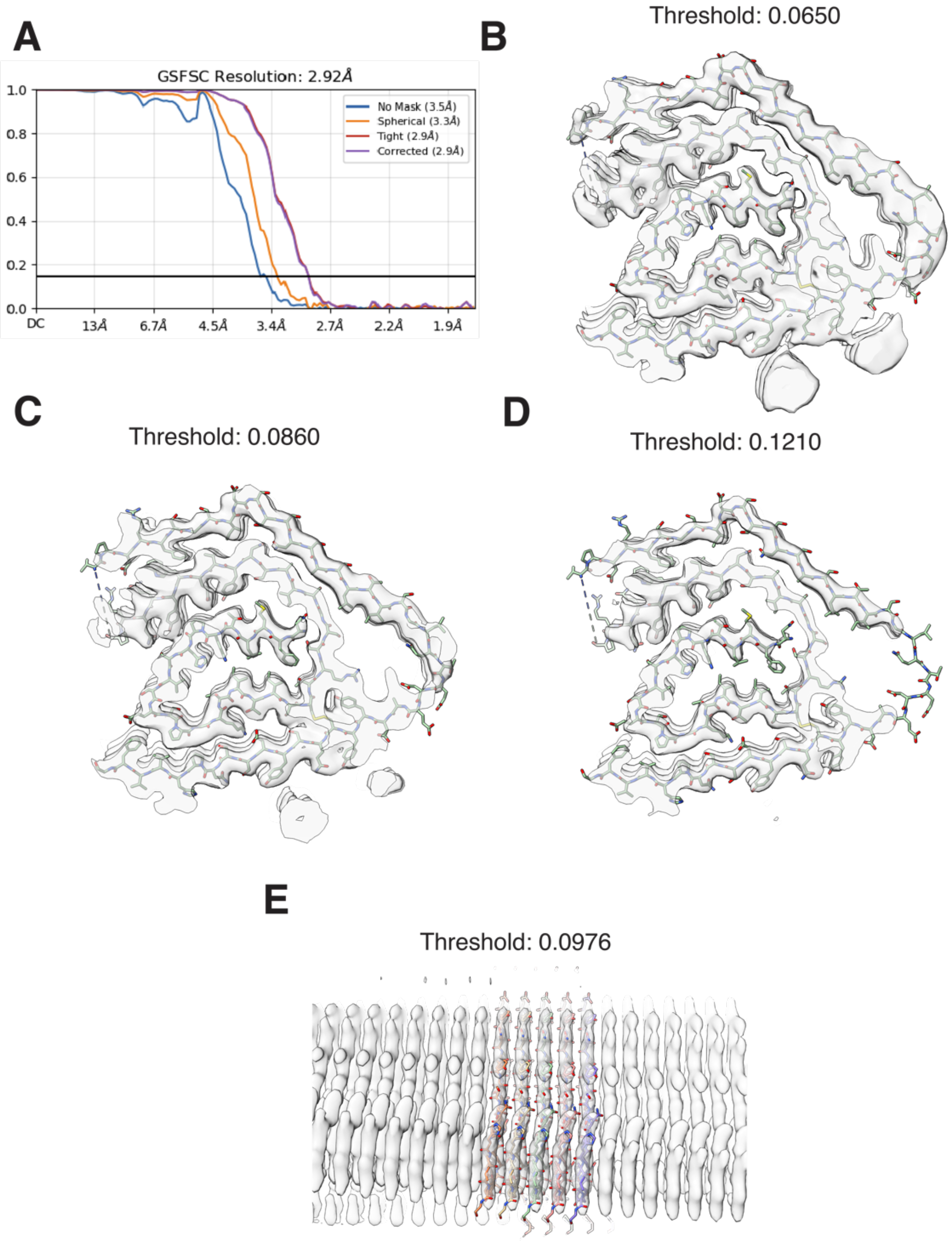

Figure S4. **Global resolution and map-model fit evaluation of amyloid structure.**
A, Fourier shell correlation (FSC) plot at a gold standard 0.143 cutoff of cardiac amyloid AL-224L.
B-E, AL-224L model superimposed over the EM map to show the map-model fit at EM map at thresholds as indicated.

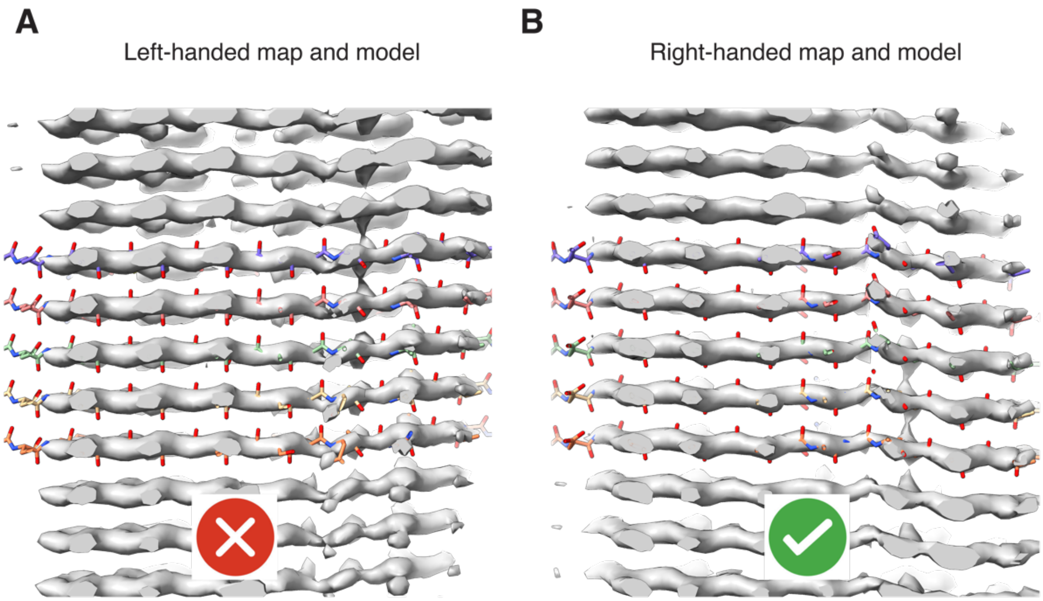

Figure S5. **Backbone corrugations reveal the AL-224L amyloid to be right-handed.**
A, Slice-view showing a superimposition of the left-handed amyloid model over the highly sharpened left-handed amyloid map. Green check icon indicates that the right-handed map and model have correct handedness. **B,** Slice-view showing a superimposition of the right-handed amyloid model over the highly sharpened right-handed amyloid map. Green check icon indicates that the right-handed map and model have correct handedness.

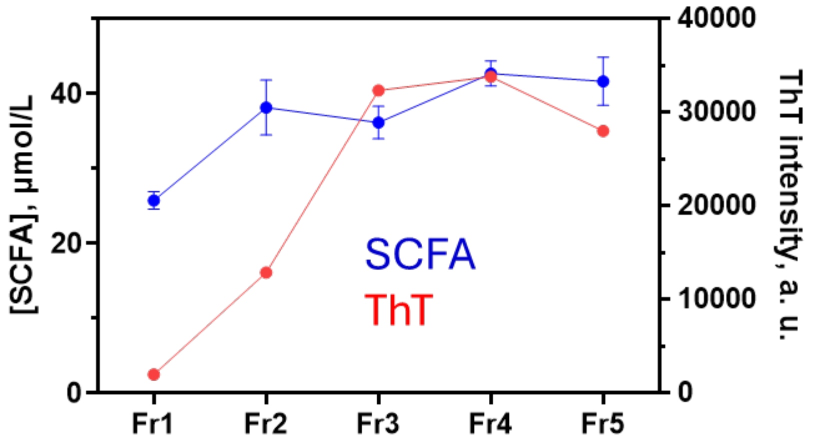

Figure S6. Amyloid and short chain fatty acid (SCFA) content in fractions 1-5 extracted from autopsied cardiac tissue AL-224.
SCFA were measured by ELISA in technical and biological duplicates; mean values ± SEM are shown. Amyloid was assessed by measuring fluorescence emission of a diagnostic dye thioflavin T (ThT) that shows increased fluorescence upon binding to amyloid. Methods provide experimental details.

SUPPLEMENTAL DATA

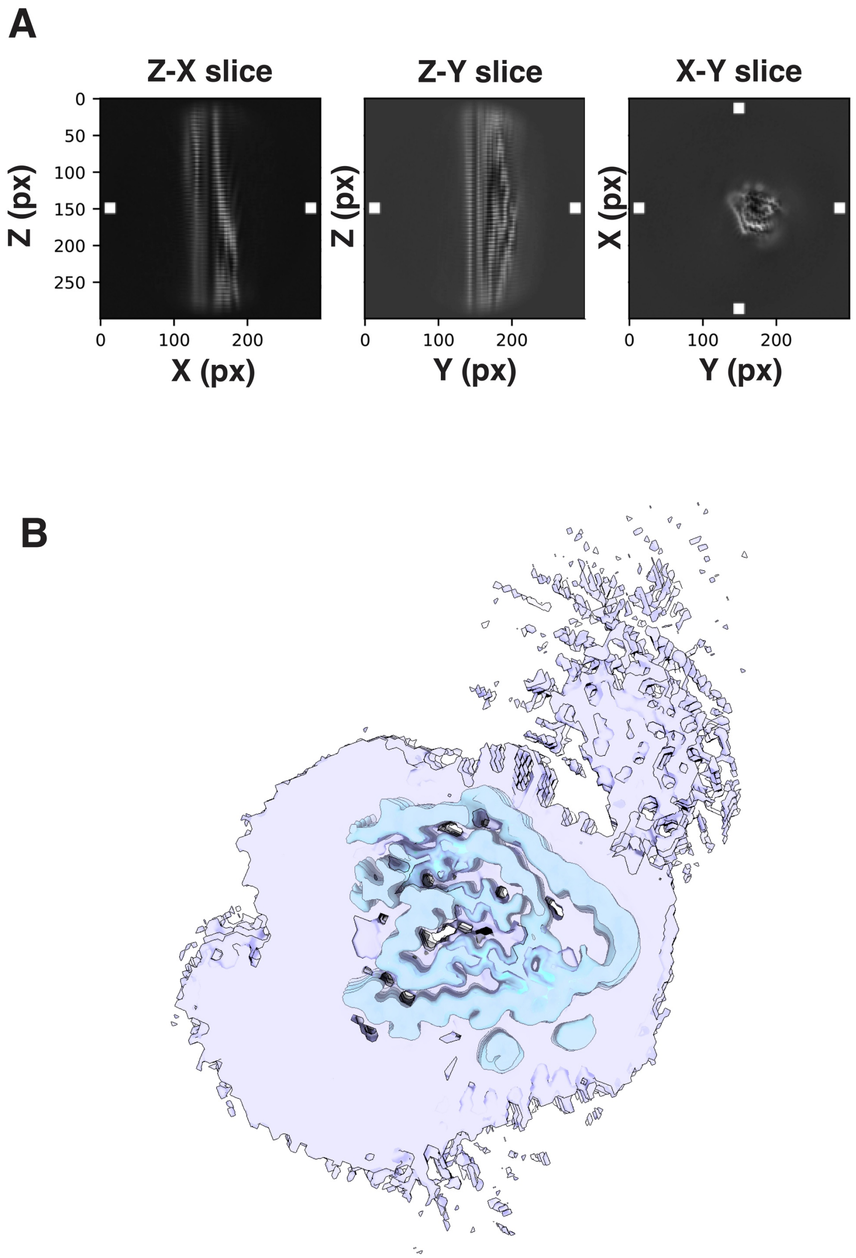

Supplemental Figure 1. **False Discovery Rate (FDR) Threshold Confidence Map.**
A, Slice-views of the unsharpened AL-224L amyloid input EM map to FDR thresholding. White boxes indicate the 3D area that was sampled to produce a noise estimate to generate a confidence map. **B,** Traditional unsharpened AL-224L amyloid EM map (blue) within an unsharpened AL-224L amyloid EM confidence map (purple) generated with FDR thresholding and viewed at a threshold of 0.99999999, equivalent to a 0.000001% false discovery rate for visible voxels.
